# Supplementary material for: New Insights into Hop Latent Viroid Detection, Infectivity, Host Range, and Transmission
Source: Viruses. 2023 Dec 23;16(1):30. doi: 10.3390/v16010030 (PMC10819085; doi:10.3390/v16010030)
Supplement: Supplementary file 1 [file viruses-16-00030-s001.zip › viruses-2718768-SI.pdf]

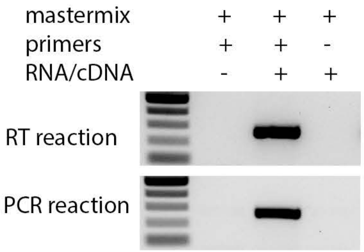

Figure S1. Does HLVd sequence secondary structure cause self-priming in RT-PCR analysis? RT-PCR analysis was performed with either RT or PCR reaction mixtures missing the primers. The upper panel shows the amplification within a reaction where primers were taken out from the RT step, while the lower panel shows the reaction amplicons where primers were missing from the PCR step.

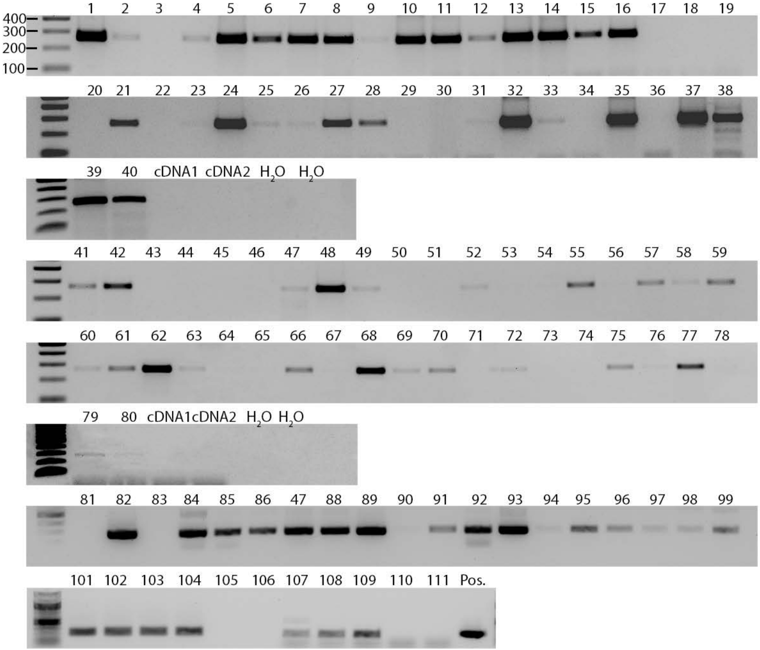

Figure S2: RT-PCR analysis of 111 hemp samples to detect HLVd. The sizes of the DNA ladder bands are shown on the left of the top panel. No RNA controls are identified as cDNA controls 1 & 2. No cDNA controls are identified as H2O. The cDNA clone of HLVd in pGEM-T Easy vector was the positive control (Pos.)

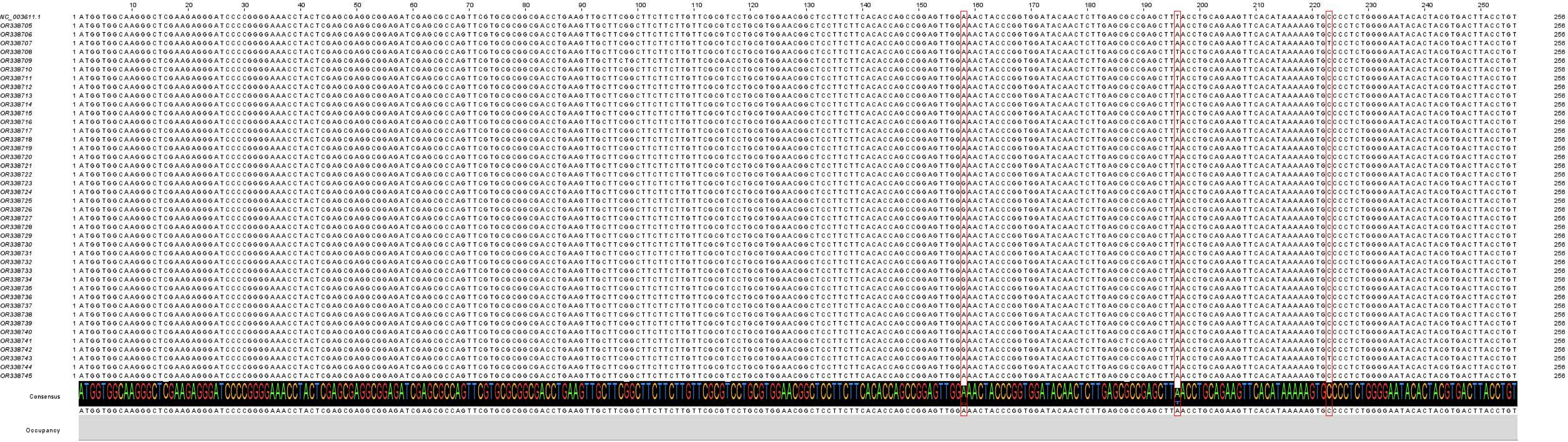
NC_003611.1
OR338705
OR338706
OR338707
OR338708
OR338709
OR338710
OR338711
OR338712
OR338713
OR338714
OR338715
OR338716
OR338717
OR338718
OR338719
OR338720
OR338721
OR338722
OR338723
OR338724
OR338725
OR338726
OR338727
OR338728
OR338729
OR338730
OR338731
OR338732
OR338733
OR338734
OR338735
OR338736
OR338737
OR338738
OR338739
OR338740
OR338741
OR338742
OR338743
OR338744
OR338745
Consensus
Occupancy

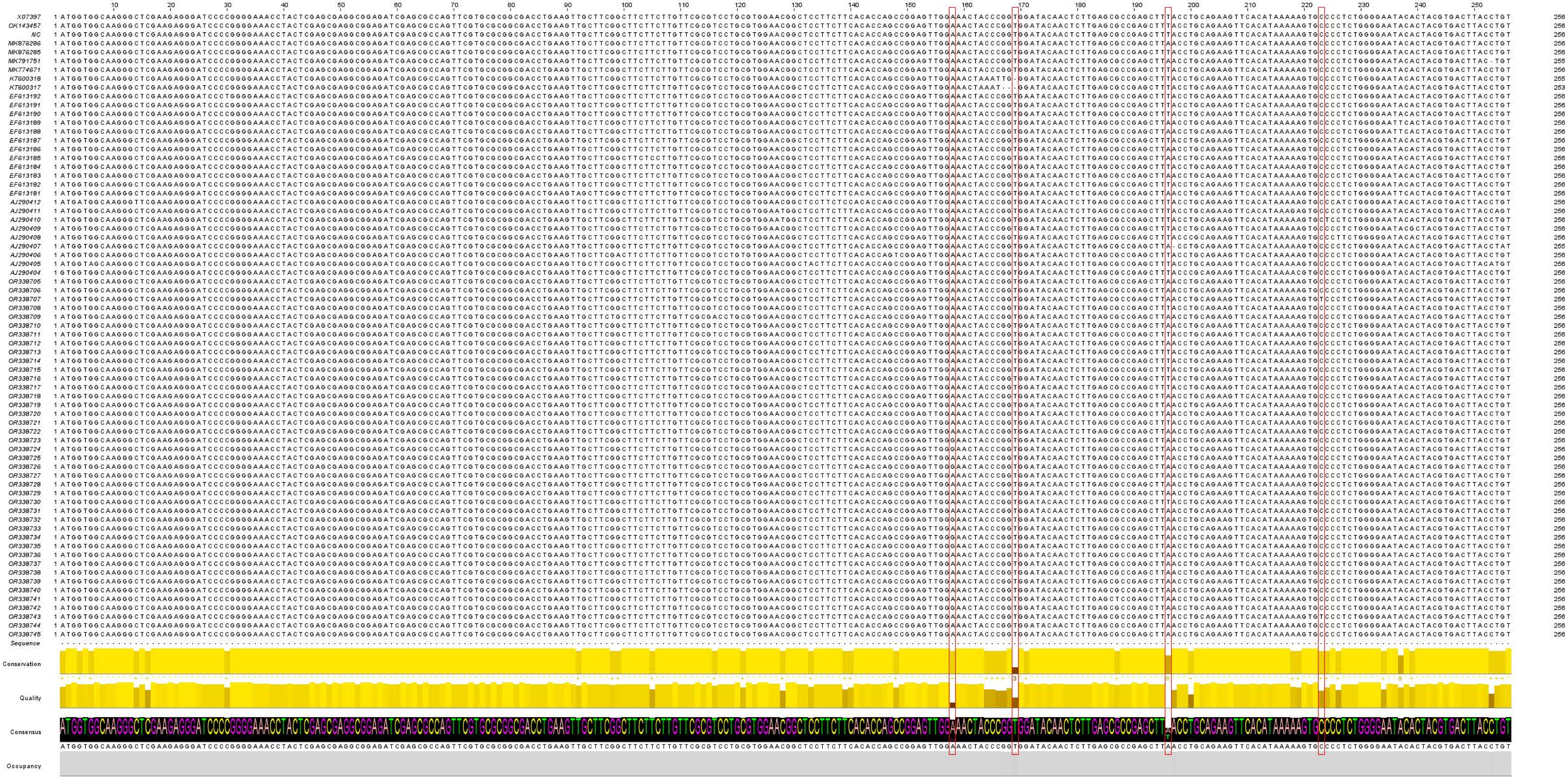
X07397
OK143457
NC
MK876286
MK876285
MK791751
MK774671
KT600318
KT600317
EF613192
EF613191
EF613190
EF613189
EF613188
EF613187
EF613186
EF613185
EF613184
EF613183
EF613182
EF613181
AJ290412
AJ290411
AJ290410
AJ290409
AJ290408
AJ290407
AJ290406
AJ290405
AJ290404
OR338705
OR338706
OR338707
OR338708
OR338709
OR338710
OR338711
OR338712
OR338713
OR338714
OR338715
OR338716
OR338717
OR338718
OR338719
OR338720
OR338721
OR338722
OR338723
OR338724
OR338725
OR338726
OR338727
OR338728
OR338729
OR338730
OR338731
OR338732
OR338733
OR338734
OR338735
OR338736
OR338737
OR338738
OR338739
OR338740
OR338741
OR338742
OR338743
OR338744
OR338745
Sequence
Conservation
Quality
Consensus
Occupancy

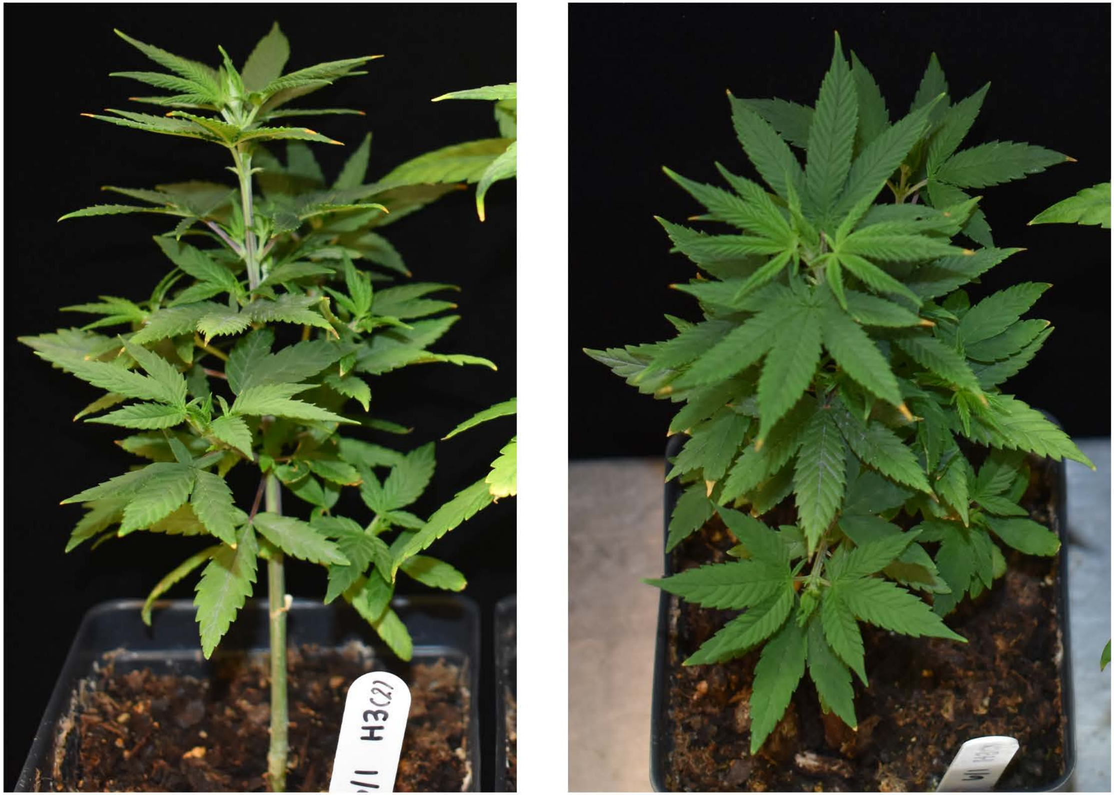

Figure S4: Side and slant view of HLVd-infected hemp. Table S1: Accession numbers of Hop latent viroid isolates recovered in this study.

Table S1. Accession numbers of *Hop latent viroid* isolates recovered in this study.

Isolate number	accession number
01	OR338705
05	OR338706
06	OR338707
07	OR338708
08	OR338709
10	OR338710
11	OR338711
12	OR338712
13	OR338713
14	OR338714
21	OR338715
24	OR338716
25	OR338717
26	OR338718
27	OR338719
28	OR338720
32	OR338721
33	OR338722
35	OR338723
37	OR338724
38	OR338725
39	OR338726
40	OR338727
42	OR338728
47	OR338729
48	OR338730
55	OR338731
58	OR338732
59	OR338733
61	OR338734
62	OR338735
66	OR338736
68	OR338737
69	OR338738
70	OR338739
72	OR338740
75	OR338741
77	OR338742
79	OR338743
80	OR338744
81	OR338745
